# Human Muscle-Derived Vascular Stem Cells Can Support Hematopoietic Stem/Progenitor Cells In Vitro

**DOI:** 10.1155/sci/4451561

**Published:** 2025-06-17

**Authors:** Tingting Yang, Jie Ma, Siqi Zhang, Rui Zhou, Xiaoping Yang, Bo Zheng

**Affiliations:** ^1^Institute of Medical Sciences, General Hospital of Ningxia Medical University, Yinchuan, China; ^2^Diagnosis and Treatment Engineering Technology Research Center of Nervous System Diseases of Ningxia Hui Autonomous Region, Yinchuan, China; ^3^Department of Hematology, General Hospital of Ningxia Medical University, Yinchuan, China; ^4^Department of Hematology, Xi'an International Medical Center, Xi'an, China; ^5^Department of Hematology, The 940th Hospital of Joint Logistic Support Force of the Chinese People's Liberation Army, Lanzhou, China; ^6^Department of Respiratory, Xi'an International Medical Center, Xi'an, China

**Keywords:** human skeletal muscle derived myoendothelial cells, human skeletal muscle-derived pericytes/perivascular cells, supporting in vitro, umbilical cord blood CD34^+^ stem cells

## Abstract

**Background:** The normal hematopoiesis of the body depends on the interaction between hematopoietic stem/progenitor cells (HSPCs) and mesenchymal stem cells (MSCs) that support the growth and development of hematopoietic cells. However, the separation of MSCs from bone marrow is somewhat limited, and the researchers have turned their attention to stromal cells outside the bone marrow. As the largest organ of human body, skeletal muscle tissue stores a variety of muscle-derived vascular stem/progenitor cells, including muscle-derived pericytes/perivascular cells (MD-PCs) and skeletal muscle derived myoendothelial cells (MECs). Studies have shown that MD-PCs and MECs are similar to bone morrow-derived MSCs (BM-MSCs), which express the surface markers of MSCs and have the potential of multidirectional differentiation. However, very few researches have been done on whether MD-PCs and MECs, like MSCs, can support HSPCs expansion/proliferation, differentiation and possible hematopoietic regulation mechanisms, so the hematopoietic support of these cells remains to be studied.

**Objective:** To identify the biological characteristics of CD146^+^ PCs and MECs isolated from human skeletal muscle and to study their supporting effect on umbilical cord blood (UCB) CD34^+^ cells in vitro.

**Methods:** Human skeletal muscle-derived CD146^+^ PCs and MECs were isolated and purified by multiparameter flow cytometry and their biological characteristics were identified. The coculture system for CD34^+^ cells with CD146^+^ PCs and MECs as trophoblastic layer, and BM-MSCs as positive control, was established in vitro, respectively. The main outcome measures, including the number and immunophenotype of the cells, the colony formation ability, the expression levels of cytokines were analyzed and compared at 1, 2, and 4 weeks after coculture.

**Results:** CD146^+^ PCs and MECs were isolated by multiparameter flow cytometry and their purity of was 92.55% ± 0.55% and 96.60% ± 1.14% (*n* = 18), respectively. Both of the cells could be differentiated into osteoblasts, chondrocytes, adipocytes, and myocytes. Compared with the positive control group of BM-MSCs, the experimental group of CD146^+^ PCs and MECs showed no significant differences in cell number, colony formation ability and immunophenotype (CD45^+^, CD34^+^ CD33^−^, CD14^+^, and CD10^+^/CD19^+^; *p*  > 0.05, *n* = 5), separately. The expression levels of cytokines in the culture supernatants of CD146^+^ PCs group, MECs group, and BM-MSCs group were measured by ELISA. The expression levels of TPO, IFN-γ, HGF, MCSF, and SCF cytokines were different among CD146^+^ PCs, MECs, and human BM-MSCs (*p* < 0.05, *n* = 3). Due to the no nourishing feeder layer in culture system, the number of CD34^+^ cells decreased significantly in the 1st week and no cells survived in the 2nd week. Therefore, the cell immunophenotype and colony analysis and the expression levels of cytokines could not be performed.

**Conclusion:** In summary, CD146^+^ PCs and MECs from human skeletal muscle, like human BM-MSCs, have hematopoietic support capacity in vitro.

## 1. Background

The hematopoietic stem/progenitor cells (HSPCs) are a group of primitive cells existing in hematopoietic tissues, which have high self-renewal ability and multidirectional differentiation potential. The self-renewal and multidirectional differentiation of hematopoietic stem cells (HSCs) depend on the hematopoietic microenvironment, which is mainly achieved by interaction with mesenchymal stem cells (MSCs), the main component of bone marrow hematopoietic microenvironment. As the precursor of bone marrow-derived mesenchymal stem cells, BM-MSCs can maintain the normal hematopoietic function of bone marrow and play an important role in hematopoietic regulation [1, 2]. MSCs secrete many hematopoietic related factors, which can promote the proliferation, differentiation, maturation, and the entry of HSPCs into the blood sinus. Currently, most of the MSCs used to promote hematopoiesis come from adult bone marrow, but factors such as material acquisition, age, and in vitro culture restrict the use of bone marrow source MSCs [3]. To change that, researchers are now looking at MSCs derived from tissue other than bone marrow. Muscle tissue is the largest tissue in the body, accounting for about 40% of the total body mass. According to relevant studies, muscle biopsy is widely accepted and obtaining muscle tissue samples is relatively easy and safe [4, 5]. Muscle-derived stem/progenitor cells (MD-SPCs) are stored in skeletal muscle tissue, these cells including satellite cells (SCs), muscle-derived stem cells (MDSCs), multipotent adult progenitor cells (MAPCs), side populations (SPs) cells, and a variety of muscle-derived vascular stem/progenitor cells, including muscle-derived pericytes/perivascular cells (MD-PCs) and skeletal muscle derived myoendothelial cells (MECs).

PCs in the capillary and microvascular walls are important components of the perivascular microenvironment, which are involved in blood flow regulation and angiogenesis, and can support the growth and reproduction of other cells and regulate the body's immune state and immune defense [6, 7]. More and more researches have showed that PCs are precursor cells of MSCs, which are similar to MSCs in cell phenotype and differentiation capability [8, 9]. Studies have shown that CD146^+^ PCs are an important part of the HSPCs Niche; in fact, CD146^+^ PCs regulate the behavior of HSPCs through intercellular contact and paracrine action and support hematopoiesis [10]. Studies by Corselli et al. [10, 11] showed that CD146^+^ PCs represented common precursor cells of MSCs and that CD146^+^ PCs, whether isolated from fat or bone marrow, were cocultured with umbilical cord blood (UCB) CD34^+^ cells for 2 weeks without the addition of hematopoietic factors; human adipose-derived CD146^+^ PCs were significantly superior to bone marrow-derived MSCs and adipose-derived CD146^−^ PCs in both amplification and support of HSPCs; CD146^+^ PCs maintain HSPCs in vitro, while CD146^−^ PCs induce HSPCs to differentiate into myeloid cells and lymphocyte.

The muscle derived MECs from human skeletal muscle were successfully isolated using multiparameter flow cytometry sorting technique (MP-FACS) by Zheng et al. [12], which expressed both markers of muscle stem cells and vascular endothelial cells (CD56^+^CD34^+^CD144^+^CD45^−^). Furthermore, studies have shown that MECs are similar to MSCs, express the surface markers of MSCs and have the potential of multidirectional differentiation into human tissue cells, such as bone tissue cells, cartilage tissue cells, and muscle tissue cells. However, it has not been reported whether MECs as trophoderm supports the expansion of HSPCs in vitro as bone marrow MSCs done. Based on the previous research of our group, we inferred that human muscle-derived vascular stem cells have a supporting role for HSCs/progenitors (hypothesis). For the first time, we will elucidate that human skeletal muscle derived CD146^+^ PCs and MECs are used as the trophoblastic layer to culture with UCB CD34^+^ cells in vitro and investigate the supporting effects of CD146^+^ PCs and MECs on the expansion of UCB CD34^+^ cells by measuring the changes in the number, immunophenotype, and colony forming ability. The results of present study will help us to find a new the rich source of stromal cells to promote HSPCs.

## 2. Materials and Methods

### 2.1. Materials

The human skeletal muscle specimens were obtained from the tissues removed during the orthopedic surgery in Ningxia Medical University General Hospital (*n* = 18). UCB was provided by obstetrics department of Ningxia Medical University General Hospital (*n* = 15). All human specimens were collected with the informed consent of the donors and their families, and the experimental program was approved by the Human Research Ethics Committee at the General Hospital of Ningxia Medical University. At the same time, the bone marrow MSCs (BM-MSCs) purchased from Saiye Biological Company of China were used as experimental controls, which was cultured in DMEM + 10% FBS + 1% P/S medium.

### 2.2. Methods

#### 2.2.1. Isolation of Mononuclear Cells in Human Skeletal Muscle

Isolation, culture, and pluripotency identification of original cells was done as previously described Zheng et al. [12]. Muscle biopsies were finely minced and digested with collagenases and dispase to obtain single cell suspension. The detail of process was as the followings: The discarded fresh muscle tissue during the operation was soaked in HBSS and the connective tissues such as fat, large blood vessels, and tendons on the muscle were removed and kept immersed in the medium (DMEM + 10%FBS + 1%P/S; Invitrogen, Carlsbad, CA, USA), and then, cut into pieces to about 1–3 mm size and move to 50 ml centrifuge tube, 1500 r/min × 5 min, discard the supernatant. The every 2.0–3.0 g muscle specimen was transfer to 30 ml of digestion solution, containing HBSS and 100 U/ml collagenase Ⅰ, 100 U/ml collagenase Ⅳ and 1.2 µg/ml dispase (all from Invitrogen), the volume of the enzyme was 300, 300, 100 μl, respectively. In a constant temperature oscillator, 37°C, digested 1–2 h, every 20 min to vigorously pipette the digested tissue 10 times with a 10 ml serological pipette, when the observation to the massive organization such as slurry digested completely, namely, add about 10 ml DMEM + 10% FBS + 1% P/S medium termination of digestion and then, centrifuged at 1500 r/min × 5 min, carefully removed the supernatant. The cell pellet was suspended in 10 ml medium, and then, filtered through a 70 µm cell strainer and centrifuged at 1500 r/min × 5 min, and the supernatant was discarded. The cells pellet was resuspended in 1 ml of erythrocyte lysis buff (eBioScience) at room temperature for 10 min, centrifuged at 1500 r/min × 5 min, discarded the supernatant, and the cells were filtered through 40 µm cell strainer and re-suspended in 1 ml of DPBS and counted finally.

#### 2.2.2. Sorting and Culturing of Human Muscle-Derived CD146^+^PCs and MECs

**Figure 1:**
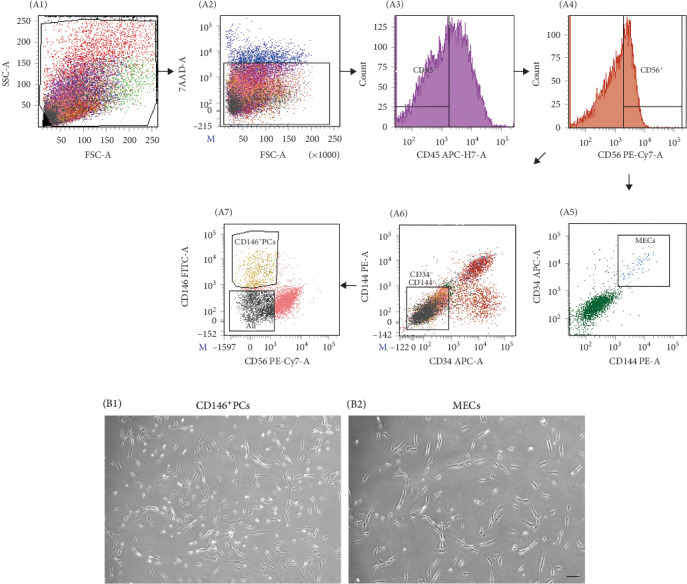
Sorting and morphological characteristics of PC cells and MECs cells. (A) Multiparameter fluorescence-activated cell sorting diagram. (A1) Following double-scatter cell population, (A2) selecting live cells according to 7-AAD, (A3) CD45^−^ cells were then selected to avoid contamination by hematopoietic cells, (A4) further separated into CD56^−^/CD56^+^, (A5) MECs (CD45^−^CD56^+^ CD34^+^CD144^+^), (A6) CD34^−^CD144^−^, (A7) CD146^+^PCs (CD146^+^CD45^−^CD34^−^CD144^−^CD56^−^). (B) Morphology of primary cultured CD146^+^ PCs and MECs in vitro. Morphology of cultured CD146^+^ PCs (B1) and MECs (B2) in P0 was analyzed under bright-field microscopy (×100). All the cells exhibited spindle-shaped morphology.

The flow antibody labeling of the cell as followings [13], including: ① the cell suspension was incubated on ice for 20–30 min at a ratio of 1:20 to the volume of mouse serum (Sigma); ② the labeled antibodies all from BD Biosciences CD34-APC, CD45-APC-H7, CD56-PE-Cy7, CD144-PE, and CD146-FITC (all 1:100) were incubated at 4°C for 20–30 min; for negative control, add equivalent concentrations of APC-, APC-H7-, PE-Cy7-, PE-, and FITC-conjugated isotype IgG antibodies (BD Biosciences) and incubate for 20–30 min. ③ Before sorting, 7-amino-actinomycin D (7-AAD, ViaProbe; BD Pharmingen) was added to each tube on ice for 10 min for dead cell exclusion. Background staining was evaluated with isotype-matched control antibodies, and a CompBeads Set (Becton-Dickinson) was used to optimize fluorescence compensation settings for multicolor analyses and sorts. Cell sorting was performed on a FACS Aria Ⅲ (Becton-Dickinson). First, CD45^+^ cells were gated out before CD56^+^ and CD56^−^ cells are separated from the CD45^−^ fraction. The CD56^+^ fraction was further subjected to CD34-CD144 selection, where only CD34^+^CD144^+^ (dual-positive) cells were marked as MECs (CD56^+^CD34^+^CD144^+^CD45^−^) and subsequently collected (Figure 1). Similarly, the CD56 fraction was further subjected to CD34-CD144 selection, and CD146^+^PCs with the phenotype of CD146^+^CD56^−^CD34^−^CD144^−^CD45^−^ were selected from the CD34^−^CD144^−^ cell population (Figure 1). Sorted cells were reanalyzed in all experiments. MECs and PCs suspension after the separation were absorbed 100 µl each, diluted to 300 µl with DPBS, and the purity of the cells after separation was back measured by the machine. The sorted cells were plated in collagen-coated 96-well plates, at a density of 500–5000 cells per well, MECs in the medium (DMEM high glucose supplemented with 10% FBS, 10% horse serum, 1% penicillin/streptomycin, 1% chick embryo extract; GIBCOBRL) and CD146^+^PCs in the medium (DMEM high glucose supplemented with 20% FBS and 1% penicillin/streptomycin, GIBCOBRL) at 37°C in a 5% CO_2_ atmosphere. At 70% confluence, cells were detached with trypsin/EDTA, replated after washing at densities between 1.0 and 2.5 × 10^3^/cm^2^ and subsequent Passage 2 cells were onto regular polystyrene culture plates/flasks further cultured for 3–4 weeks for further experiments and summarized in Table 1.

### 2.3. Phenotypic Analyses of CD146^+^ PCs and MECs by Flow Cytometry

Culture and identification of MSCs were elaborately explained in the research by Fathi et al. [14, 15]. Preparation of CD146^+^PCs and MECs cell suspensions P3–8, and then were add CD105, CD90, CD73, CD44, CD45, CD34, CD31, CD14, and HLA-DR, monoclonal antibodies, respectively, for labeling and set up blank control and the isotype-matched control antibodies, detection, and analysis. At the same time, BM-MSCs were used as control cells to be labeled and analyzed.

### 2.4. In Vitro Osteogenesis, Adipogenesis, Chondrogenesis, and Myogenesis Differentiation Assay

In vitro differentiation assay of CD146^+^ PCs and MECs at passage 3–8 were performed in the plate at a density of (1.0 − 3.0) × 10^4^/well in 5% CO_2_, 37°C saturated humidity incubator, respectively, and each experimental group and control group were set three wells at the same times for the following experiments.

### 2.5. Osteogenesis and Adipogenesis-Induced Differentiation

MECs and CD146^+^PCs of P3−8 were planted in 24-well plate at the density of (1.0 − 3.0) × 10^4^/well; the experimental group and control group were set with three well in MECs medium and PCs medium, respectively, and placed in a saturated humidity incubator with 5% CO_2_ at 37°C. After 24 h, when the cells reached 60%–80% confluent, the media of MECs and PCs positive group were replaced with osteogenesis induction medium (Gibco) or adipogenic induction medium (Gibco), respectively, and the media were changed every 3 days; after 14 days, calcium was detected with alizarin red kits (Solarbio) and lipids was detected with oil red O kit (Solarbio) at RT, respectively.

### 2.6. Induction of Chondrogenic Differentiation

MECs and CD146^+^ PCs of P3−8 were planted in a 12-well plate at a density of 2 × 10^5^/microsphere, each microsphere was seeded with 10 µl cells, and a spherical cell mass was formed after 24 h in the induction group, the cartilage induction medium was added, and in the control group, MECs and PCs media were added, and the media were changed every 3 days. After induction for 28 days, the cartilage pellets were collected and frozen sections (thickness 8 µm) after embedding in OCT Compound and then Alcian Blue staining was performed.

### 2.7. Myogenic Induction of Differentiation

MECs and CD146^+^ PCs of P3−8 were planted in a six-well plate at a density of 1.0 × 10^5^/well and the media of MECs and CD146^+^ PCs were added, respectively. When the cell fusion reached about 80% after 24 h, the medium in the induction group was changed to fusion medium (DMEM + 1% FBS + 1% HS), and the control group continued to add MECs and CD146^+^ PCs medium, and the medium was changed every 3 days. After 10 days of culture, immunofluorescence staining was performed, fixed with 40 g/l paraformaldehyde for 1 min, washed twice with HBSS, permeated with 0.2% Triton for 10 min, cleaned with HBSS two times, 5% HS blocking for 1 h. Then, monoclonal anti-myosin (Skeletal, Fast) antibody (Sigma corporation, USA) produced in mouse incubated at room temperature for 2 h, and washed twice by HBSS; biotinylated goat anti-mouse IgG (Vector Laboratorie, USA) was incubated at room temperature for 1 h and washed twice with HBSS; streptavidin and Alexa Fluor555 conjugate (Invitrogen Company, USA) were incubated at room temperature for 1 h and washed with HBSS twice; DAPI (Sigma corporation, USA) stained nuclei for 5 min and analyzed it with a fluorescent microscope.

### 2.8. Isolation and Purification of CD34^+^ Cells From Human UCB

Fresh fetal umbilical blood was collected by obstetrics department and mononuclear cells were extracted with Ficoll–Paque separation solution, and CD34^+^ cell immunomagnetic bead sorting was conducted according to Human CD34 MicroBead Kit. Flow cytometry was used to detect the positive rate of CD34^+^ cells.

### 2.9. In Vitro Coculture of Stromal Cells and UCB CD34^+^ Cells

#### 2.9.1. Establishment of In Vitro Amplification System

The coculture procedure has been previously described by Fathi et al. [16]. Experimental groups, including CD146^+^ PCs and MECs, were used as trophoblastic layer respectively. BM-MSCs as trophoblastic layer were set as positive control group and negative control group was no trophoblastic layer. CD146^+^ PCs, MECs, and BM-MSCs of the P3−8 generation were inoculated into 96-well collagen-coated plates with 1.5 × 10^4^/well, and their respective medium was added. After 24 h, the cells were completely attached to the wall, and treated with 4 mg/l mitomycin for 2 h. Then, HBSS was added to the cells and washed for three times, and the medium was added and placed in an incubator. After 24 h, CD34^+^ cells of freshly prepared UCB were inoculated on trophoblast cells at a density of 5 × 10^4^/well, and CD34^+^ cells without trophoblastic layer were directly inoculated as the negative control group. Three secondary pores were set in each group, and the coculture medium (RPMI 1640 + 5% FBS + 1% P/S) was added, and the incubator was placed.

#### 2.9.2. Flow Cytometric Analysis of Cultured UCB CD34^+^ Cells

Analyze the immunophenotype of cells after coculture, after 1, 2, and 4 weeks of coculture, UCB cells were collected and counted. The single-cell suspension with cell concentration of 1 × 10^5^/ml was adjusted to 200 µL, and 1 µl each of CD34-APC, CD45-APC-H7, CD33-FITC, CD19-PE-CY7, CD10-PE, and CD14-Alexa 700 was added into the sample tube. At the same time, the same ectype control was set up. 7-AAD ViaProbe was added before entering the machine for flow cytometry analysis.

#### 2.9.3. Colony Forming Unit Assay

For CD34^+^ cell colony formation and morphological identification of blood cells after coculture, the cells from experimental group and positive control group were taken and 3 × 10^3^ cells of each group into 3 ml MethoCult H4535 Enriched without EPO semisolid medium (Stemcell Cell Technologies), then, 1 ml cell mixture was set in 35-mm diameter Prtri dish (total three set for each group) under humidified 5% CO_2_ atmosphere at 37°C. After colony formation at 14 days, the colony of granulo-macrophage colony forming unit (CFU-GM) was observed under a microscope, and then, transferred to the EP tube containing 50 µl HBSS and repeatedly stirred. The cells were recovered and cytospun onto slides. Swiss Giemsa staining was performed to examine cell morphology. After the slides were air-dried, the morphology of the cells was observed under an inverted fluorescence microscope.

#### 2.9.4. The Expression Level of Cytokines in the Culture Supernatant was Detected by ELISA

Immunocytochemistry was previously reported by Farahzadi et al. [17]. The supernatant of the coculture was harvested, centrifuged at 1500 rpm × 5 min, and the cytokine detection of TPO, VEGF, IFN-γ, TNF-γ, HGF, MCSF, SCF, IL-6, and IL-3 was analyzed by ELISA technique in a microplate reader, according to the kit manufacture (Cloud-Clone Corp, USA).

### 2.10. Statistical Analysis

SPSS 26.0 software was used to perform statistical analysis. The mean of measurement data was ±standard error ($\overset{―}{\chi }\pm S_{\overset{―}{\chi }}$). Comparisons between two groups were analyzed using independent two-tailed Student's *t* tests, while comparisons between more than two groups were analyzed using one-way ANOVA. *p* values <0.05 were considered statistically significant.

## 3. Results

### 3.1. Sorting and Identification of PC and MECs Cells

Human skeletal muscle mononuclear cells were isolated from 18 human skeletal muscle tissues, including 10 males and eight females, aged from 19 to 68 years. The mass of isolated muscle was 4.75 ± 1.04 g and mononuclear cells were (2.70 ± 0.41) × 10^6^ per gram of muscle (*n* = 18). The FACS parameters were corrected by flow antibody multi-parameter labeling and the target cell population was depicted (Figure 1A A1), live cells were selected according to 7-AAD (Figure 1A A2), and MECs cell population: CD45^−^CD56^+^CD34^+^CD144^+^ (Figure 1A A5), and CD146^+^ cell population, where PCs: CD146^+^CD45^−^CD34^−^CD144^−^CD56^−^ was selected (Figure 1A A7). The positive rate of MECs was 0.04% ± 0.01%, the cell number was (1.26 ± 0.32) × 10^4^, the positive rate of PCs was 0.32% ± 0.09%, the cell number was (3.36 ± 0.66) × 10^4^ (Table 2). The purity of CD146^+^PCs and MECs were measured by FACS. The purity and survival rate of CD146^+^PCs were 92.55% ± 0.55% and 95.25% ± 0.77%, respectively, and MECs were 96.60% ±1.14% and 95.68% ± 1.20% (Table 2). The morphology of the cells was observed under inverted phase contrast microscope. The newly separated cells showed spherical shape, bright cytoplasm, and evenly distributed in the culture plate. At about 5–7 days, a small number of short spindle, triangle, and irregular cells adhered to the wall, and the primary cells grew slowly, and the density of cells gradually increased to 80% at 10–14 days. CD146^+^ PCs and MECs cells were cultured to P8, and the cells were in good condition with regular morphology. CD146^+^ PCs were spindle cells with long ends, enlarged in the center of cell body, uniform nucleus, and small nucleus (Figure 1B B1). MECs cells were shorter than CD146^+^ PCs cells, with spindle and triangular shapes and irregular polygons (Figure 1B B2).

### 3.2. PCs and MECs Present Similar Characteristics of MSCs

**Figure 2:**
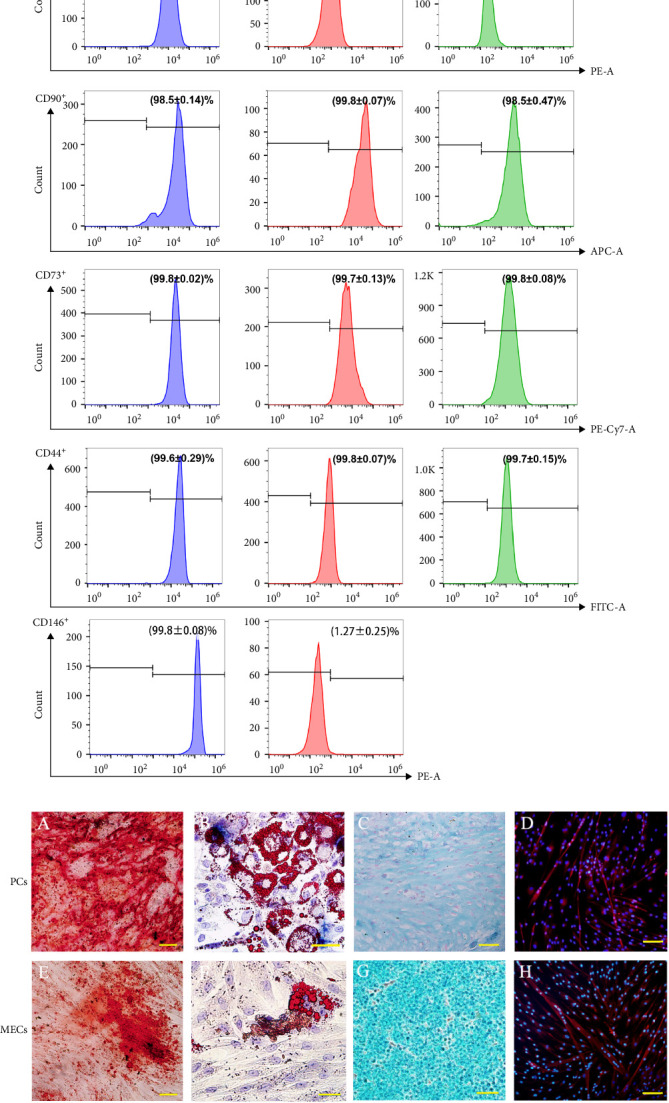
Identification of stem cell properties of PCs cells and MECs cells. (A) Immunophenotypes of CD146^+^ PCs and MECs cells with BMMSCs as control analyzed by flow cytometry. (B) Multipotency of CD146^+^PCs and MECs, (A, E) osteogenic induced differentiation showed a positive reaction (alizarin red staining, ×100), (B, F) adipogenic differentiation showed an amount of red lipid droplets (oil red O staining, ×50), (C, G) chondrogenic induced differentiation showed the positive reaction (Alcian blue staining, ×200), (D, H) myogenesis differentiation showed the myotube formation and express fast MyHC (red; immunofluorescence staining, ×100).

In order to determine whether PCs and MECs have the characteristics of MSCs, we carried out immunophenotypic analysis and multipotential induction differentiation experiments, and finally obtained the following results. Immunophenotypic analysis of the cells, both PCs and MECs highly expressed MSC surface antigens as followings, CD146^+^ PCs expressed CD105 99.5% ± 0.25%, CD90 98.5% ± 0.14%, CD73 99.8% ± 0.02%, CD44 99.6% ± 0.29%, and CD146 99.8% ± 0.08% (*n* = 8). MECs expressed CD105 99.6% ± 0.11%, CD90 99.8% ± 0.07%, CD73 99.7% ± 0.13%, and CD44 99.8% ± 0.07% (*n* = 6), and with low expression CD146 1.27% ± 0.25% (Figure 2A). Neither of them expressed CD45, CD34, CD31, CD14, and HLA-DR (data not show). The above results showed that the CD146^+^ hMD-PCs and MECs obtained by flow cytometry were the same as those BM-MSCs (Figure 2A). In terms of multipotential induction differentiation: ① Osteogenic induced differentiation: Calcium deposits were observed on cell surface in CD146^+^ PCs and MECs groups and no calcium deposits were observed around cells in control group (Figure 2B A, E). ② Adipogenic induced differentiation: A large number of red lipid droplets were accumulated in the cytoplasm of CD146^+^ PCs and MECs groups, while no lipid droplets were formed in the control group (Figure 2B B, F). ③ Chondrogenic induction and differentiation: Cysts and lacunae of cartilage were observed in CD146^+^ PCs group after frozen section staining and the nuclei in cysts of cartilage were light red (Figure 2B C). In MECs group, chondrogenic cartilage induced differentiation was round and blue globules and no obvious cysts, cysts and nuclei of cartilage were observed (Figure 2B G). Cells in the control group showed milky yellow, loose, soft, and fragile masses, which failed to form cartilage globules (data not shown). ④ Myoblast induced differentiation: Myosin heavy chain immunofluorescence staining was performed in CD146^+^ PCs and MECs groups and multiple typical fusiform multinucleated myotube formation was observed in both groups, with at least three to five fine nuclei in one myotube, while no obvious multinucleated myotube formation was observed in the control group (Figure 2B D, H).

### 3.3. CD146^+^ PCs and MECs Promote the Proliferation and Colony Formation of HSPCs

**Figure 3:**
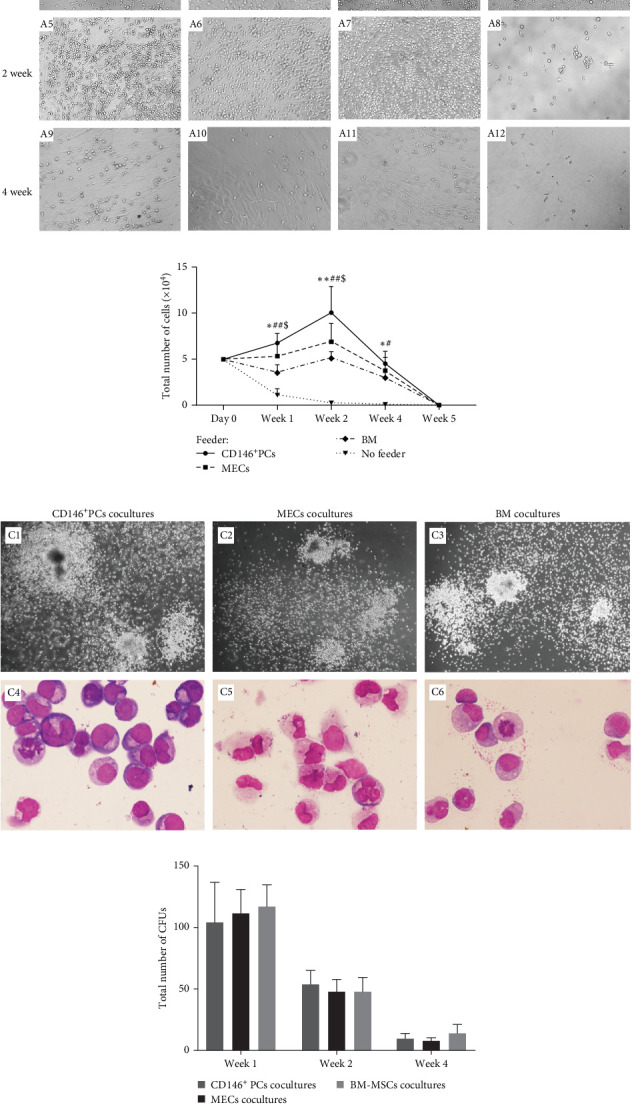
The proliferation and colony-forming ability of hematopoietic cells were evaluated after co-culture. (A) Morphology of UCB CD34^+^ cells after coculture in vitro, (A1–A4) The cell morphology of each experimental group for 1 week, (A5–A8) the cell morphology of each experimental group for 2 weeks, and (A9–A12) the cell morphology of each experimental group for 4 weeks (×100). (B) Compared to no feeder layer group, CD146^+^ PCs, MECs, and BM-MSCs as trophoblasts efficiently expanded UCB CD34^+^ HSPCs cells in vitro (*n* = 5; *⁣*^*∗*^*p*  < 0.05, *⁣*^*∗∗*^*p*  < 0.01, vs. CD146^+^PCs group; ^#^*p*  < 0.05, ^##^*p*  < 0.01, MECs group; and ^$^*p*  < 0.05 vs. BM-MSCs group). All data are presented as mean ± SEM. (C) Morphology and colony formation ability of umbilical cord blood CD34^+^ cells in each culture system, (C1–C3) The morphology of granulocyte-macrophage colonies was observed microscopically after coculture for 14 days (×100), (C4–C6) the blood cells from colonies in each culture system were the stained by Swiss Gimza and observed under a microscope (×1000). (D) The number of colonies decreased gradually at 1, 2, and 4 weeks, and the difference was not significant among CD146^+^ PCs, MECs, and BM-MSCs at any of the three time points (*p* > 0.05, *n* = 5).

In order to determine whether CD146^+^ PCs and MECs can promote the proliferation and differentiation of HSPCs, this study established three coculture systems without adding any exogenous cytokines and evaluated the support effect of CD146^+^ PCs and MECs on HSPCs by detecting the number of CD34^+^cells, colony forming ability, and immunophenotype after coculture in vitro. CD146^+^ PCs, MECs, and BM-MSCs as control were used as stromal cells. Through direct contact coculture method, UCB CD34^+^ cells were observed under a microscope to form small and bright circular suspension cells floating in the medium. After 1 week observation, the experimental group (CD146^+^PCs and MECs as trophoblast layer) and the positive control group (BM-MSCs as trophoblast layer) were observed. The stromal cells did not change the morphology and the CD34^+^ cells were larger cell size, with good circular refraction and the cell density increased compared with the negative control group (no trophoblast group; Figure 3A A1–A4). After 2 weeks observation, the experimental group and the positive control group showed obvious amplification of UCB CD34^+^ cells with good morphology, full cytoplasm, and bright cytoplasm. The number of cells in the negative control group was significantly reduced, scattered, poor refraction, irregular cell morphology, and dead cell masses (Figure 3A A5–A8). At 4 weeks, the number of cells in the experimental group and the positive control group was significantly reduced, with a small number of viable cells, poor refraction of fine substance, irregular cell morphology, and no viable cells in the negative control group (Figure 3A A9–A12).

We calculated the number of UCB CD34^+^ cells at different time points in each culture system to determine whether CD146^+^PCs and MECs promote the proliferation of HSPCs. The inoculation density of UCB CD34^+^ cells in the four coculture systems was 5 × 10^4^, respectively. The number of UCB CD34^+^ cells increased slightly after 1 week in the culture system with CD146^+^PCs, MECs, and BM-MSCs as the trophoblast layer and the number of UCB CD34^+^ cells peaked after 2 weeks. With the extension of coculture time, the number of UCB CD34^+^ cells in the experimental group and positive control group decreased gradually and died at the 5 weeks. UCB CD34^+^ cells with no stromal cell group decreased significantly during culture and remained cell-free until 4 weeks. After statistical analysis, there was no statistically significant difference in the number of UCB CD34^+^ cells among CD146^+^ PCs, MECs and BM-MSCs as trophoblasts and had no significant difference at 1, 2 and 4 weeks (*p*  > 0.05, *n* = 5, Figure 3B). Compared with experimental and positive control groups, the number of cells in the blank group formed varied at 1, 2 and 4 weeks, this difference was significant (*p*  < 0.05, *n* = 5, Figure 3B).

The colony-forming ability reflects the ability of UCB CD34^+^ cells to produce hematopoietic progenitor cells (HPCs) after culture. After 1, 2, and 4 weeks of coculture the cells were harvested from the coculture system, and then, the cells were plated in methylcellulose medium and colony formation was visible after 7–10 days of culture. After 14 days of culture, CFU granulocyte-macrophage with more than 50 cells were observed and counted under the microscope (Figure 3C C1–C3). The colonies from each culture system were individually picked, stained by Swiss Giemsa. The cell morphology was observed under the microscope and blood cells such as macrophages, granulocytes, and monocytes could be observed with clear cell outline, deep nuclear coloration, and clear cytoplasm (Figure 3C C4–C6). The number of colonies was the highest in each group at the first week of coculture and gradually decreased with the extension of coculture time. The data showed that there were no significant statistical differences in the number of colonies among CD146^+^ PCs, MECs, and BM-MSCs (*p* > 0.05, *n* = 5) in 1, 2, and 4 weeks (Figure 3D). The blank control group did not undergo colony culture, because the number of cells in the blank control group decreased significantly when cocultured for 1 week, and almost no cells existed by 2 weeks.

### 3.4. CD146^+^ PCs and MECs Promote the Differentiation and Directional Maturation of HSPCs, Have a Supportive Effect In Vitro

**Figure 4:**
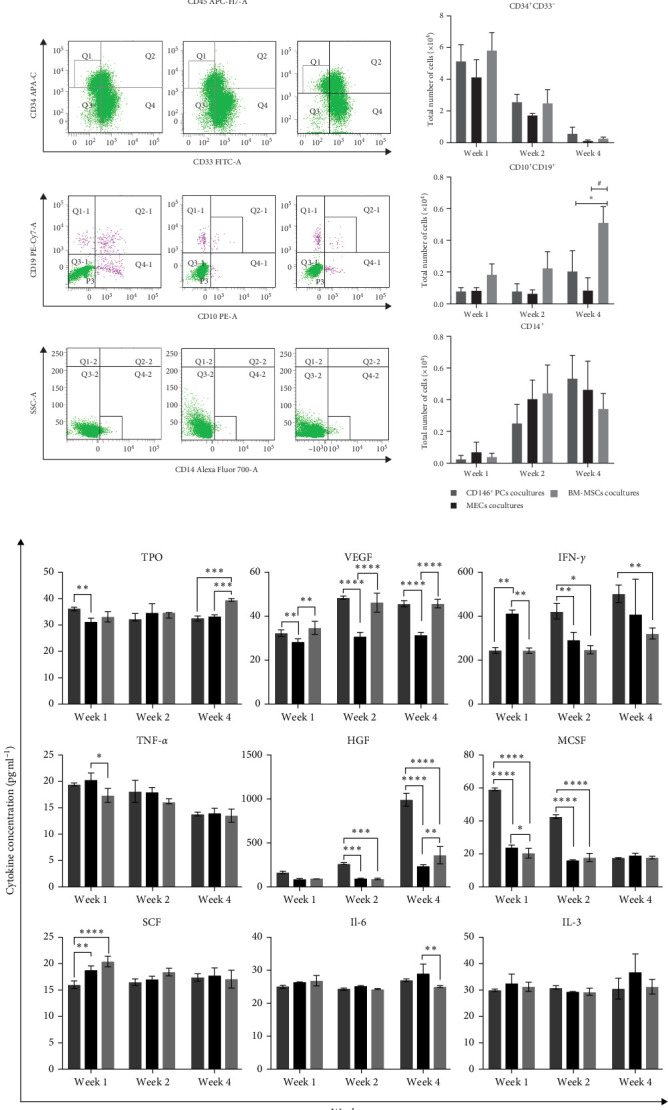
Flow detection and phenotypic statistical analysis of hematopoietic cells after coculture and expression level of cytokines in supernatant. (A) Top to bottom, flow cytometric plots of CD45^+^, CD34^+^CD33^−^, CD10^+^CD19^+^, and CD14^+^ phenotypes after coculture with CD146^+^PCs, MECs, and BM-MSCs in the culture systems at 1, 2, and 4 weeks. CD45^+^ expression rate in the CD146^+^PCs group was higher than that of the positive control group (BM-MSCs) at the 2nd week, and CD10^+^/CD19^+^ expression rate in CD146^+^PCs group and MECs group was lower than that of the BM-MSCs group during the 4th week of culture, with statistically significant differences (*p* < 0.05, *n* = 5). (B) The expression levels of the cytokines in the coculture supernatants were measured by ELISA at 1, 2, and 4 weeks. (*⁣*^*∗*^*p* < 0.05, *⁣*^*∗∗*^*p* < 0.01, *⁣*^*∗∗∗*^*p* < 0.001, *⁣*^*∗∗∗∗*^*p* < 0.0001, *n* = 3). All data are presented as mean ± SEM.

The hematopoietic cell markers of the cultured cells were analyzed by FACS detection after UCB CD34^+^ cells were cocultured with each of CD146^+^ PCs, MECs, and BM-MSCs as trophoblast in the culture systems for 1, 2, and 4 weeks, respectively. Monoclonal antibodies recognizing the following cell-surface markers were used for flow cytometry, including CD45 (whole blood cells), CD34/CD33 (HPC), CD10/CD19 (lymphocytes), and CD14 (myeloid cells). The expression of these markers was measured to compare the similarities and differences of the hematopoietic cell ability of each culture system. The cells from each coculture system at 1, 2, and 4 weeks were collected for enumeration, and the expression of CD45^+^, CD14^+^, CD10^+^/CD19^+^, and CD34^+^CD33^−^ in UCB CD34^+^ cells was determined by flow cytometry. The CD45^+^ expression rate of UCB CD34^+^ cells in the CD146^+^ PCs group was higher than that of the positive control group (BM-MSCs) at the second week of coculture, and the CD10^+^/CD19^+^ expression rate of the cells in CD146^+^PCs group and MECs group was lower than that of the BM-MSCs group during the 4th week of culture, with statistically significant differences (*p* < 0.05, *n* = 5). There were no significant differences in the expression of CD34^+^CD33^−^, CD14^+^, and CD34^+^ immunophenotypes of the other subtypes at 1, 2, or 4 weeks in each group (*p* > 0.05, *n* = 5). The cell immunophenotype could not be done because the trophoblast-free UCB CD34^+^ cells had few cells counted at 1 week and all died at 2 weeks, and the cell count failed to do the flow analysis (Figure 4A).

Hematopoietic cytokines play a pivotal role in promoting the differentiation, proliferation, and directional maturation of HSCs both in vitro and in vivo. With the aim of evaluating the effects of coculture on cytokine secretion, we collected the supernatants of different culture systems after 1, 2, and 4 weeks of coculture. The results showed that the TPO level in the CD146^+^ PCs group was significantly higher compared with the MECs group at 1 week. Meanwhile, TPO levels were significantly lower in CD146^+^ PCs and MECs compared to BM-MSCs at 4 weeks (Figure 4B). At 1, 2, and 4 weeks, VEGF levels were considerably higher in CD146^+^ PCs and BM-MSCs than in MECs. At 1 week, CD146^+^ PCs and BM-MSCs had significantly lower levels of IFN-γ than the MECs group. However, at 2 and 4 weeks, the CD146^+^ PCs group had significantly higher levels of IFN-γ compared with both the MECs and BM-MSCs groups. The level of TNF-α in BM-MSCs group was significantly lower than that in MECs at 1 week. The concentration of HGF in the CD146^+^ PCs group was observed to be significantly higher when compared to the MECs and BM-MSCs groups at 2 and 4 weeks. Meanwhile, HGF level in BM-MSCs group was significantly higher than that in MECs at 4 weeks. The MCSF level in CD146^+^ PCs was found to be significantly higher than that in MECs and BM-MSCs at 1 and 2 weeks. Meanwhile, MCSF level in MECs group was significantly higher than that in BM-MSCs at 1 week. The SCF level of CD146^+^ PCs group was significantly lower than that of MECs and BM-MSCs groups at 1 week. IL-6 level in MECs group was significantly higher than that of BM-MSCs at 4 weeks. The statistical analysis revealed that the IL-3 expression levels did not exhibit a significant difference among the various groups at 1, 2, and 4 weeks.

## 4. Discussion

HSCs (HSPCs) are a group of pluripotent stem cells with high self-renewal ability and ability to differentiate into various types of mature blood cells that exist in hematopoietic tissues [18]. HPCs are progenitor cells that are derived from HSCs that proliferate and differentiate into various types of blood cells in a specific microenvironment. The first demonstration of the existence of HSCs was reported in 1961 by Till and McCulloch. They discovered the regeneration potential of single bone marrow cells, thus, confirming the existence of multipotent hemopoietic stem cells [19]. Since the first successful implementation of HSC transplantation (HSCT) for leukemia by Thomas' team in the 1950s [20], HSCT has become an important means to treat various benign and malignant hematological diseases such as leukemia, myeloma, severe aplastic anemia, genetic or immune defects, and bone marrow failure caused by cancer chemotherapy [21]. HSPCs can be derived from bone marrow, mobilized peripheral blood, UCB, and placenta [22]. The earliest clinical application of HSCT was a bone marrow transplantation treatment requiring a matched donor-recipient. Research shows that the number of HSPCs transplanted is closely related to the success rate of transplantation, immune recovery time, and survival rate [22]. However, bone marrow harvesting is invasive and limited; peripheral blood contains less than 0.1% HSPCs, and granulocyte colony-stimulating factor is used to mobilize and release HSPCs from bone marrow into peripheral blood, which often requires multiple mobilizations due to insufficient numbers of HSPCs and poor mobilization [20]. Compared with bone marrow and peripheral blood, UCB has the advantages of wide source, convenient collection, low immunogenicity, and low incidence of graft-versus-host disease. Thus, UCB serves as an important source of HSCs [23]. The absolute number of HSPCs in a single UCB sample is insufficient, which limits the transplantation needs of adult patients and overweight children [24]. How to effectively expand the number of HSPCs in vitro, improve their implantation rate, and shorten hematopoietic reconstitution is still a hot and difficult research topic and has important clinical and scientific significance.

CD34 is the earliest and the most commonly used surface molecule for hematopoietic cell enrichment in basic or clinical research. In the process of HSCT, CD34 is expressed in most of the cells that can make the hematopoietic reconstitution of myeloablative recipients. Of course, clinical autologous or allogeneic hematopoietic cell transplantation also proves the implantation ability of human CD34 positive cells [25]. The commonly used methods for the expansion and culture of HSPCs in vitro include cytokine amplification technology, small molecule compound amplification technology, and stromal cell coculture expansion technology [26, 27]. The amplification of HSCs requires the combined application of different cytokines, but the optimal combination and concentration of cytokines have not been determined. Although molecular compounds have made a major breakthrough in improving the expansion of HSCs, their safety still needs to be further verified.

It is well known that the proliferation, differentiation and development of hematopoietic cells depend on the bone marrow hematopoietic microenvironment in which they are located. Stromal cells provide a natural microenvironment for the proliferation, self-renewal, and differentiation of HSCs through the interaction between cells and the secretion of cytokines [28]. The in vitro culture system of HSCs and stromal cells coculture aims to promote the proliferation of HSCs by simulating the hematopoietic microenvironment [29]. Song et al. [30] believed that coculture of HSCs and bone marrow MSCs (BM-MSCs) can preserve the long-term expansion ability of HSCs in vitro for several weeks without adding cytokines. Zhou et al. [31] used the perivascular cells isolated from human adipose tissue as the feeder layer of CD34^+^ cells in cord blood. Without adding exogenous cytokines and growth factors, CD146^+^ hAD-PCs can expand CD34^+^cells and CD34^+^ cells can be maintained for at least 4 weeks. de Lima et al. [32] found that UCB cells cocultured and expanded with MSCs in vitro were transplanted in vivo, which shortened the recovery time of neutrophils and platelets after adult transplantation [33]. In addition, the study also showed that stromal cells are also important to avoid HSC differentiation.

In 1978, Deans and Moseley [34] proposed the concept of “niche,” which made people realize that the bone marrow microenvironment is very important to the biological characteristics of HSCs. It can regulate the function and differentiation of HSCs. HSCs in the niche can maintain self-renewal and differentiate into HPCs once they leave the niche [35]. Niche is the internal microenvironment of bone marrow, which is mainly composed of osteoblasts, MSCs, endothelial cells, and other cells. It produces specific microenvironment in hematopoietic tissue through cell secretion factors, expression of molecules combined with HSCs, and production of extracellular matrix, which provides an important prerequisite for self-renewal, differentiation, and survival of HSCs [36]. Therefore, the development of an in vitro system that can maintain the hematopoietic niche microenvironment will improve the expansion strategy of HSPCs in vitro. Studies have shown that the presence of MSCs in the bone marrow microenvironment is the key to inducing self-renewal of HSCs. MSCs can secrete a variety of cytokines and express adhesion molecules to promote self-renewal and differentiation of HSCs.

MSCs are a type of stem cells with multidirectional differentiation capacity in the early developmental mesoderm, which have long-term self-renewal potential and multidirectional differentiation ability. MSCs were first isolated experimentally by Friedenstein [37] in the 1960s and are characterized by their multidirectional differentiation potential, support for hematopoiesis and promotion of HSCs implantation, and regulation of immunity. Such cells were discovered from long-term cultures of bone marrow stromal cells that had grown adherently and differentiated into osteoblasts and stromal cells using a natural anchorage method. MSCs do not yet have specific surface antigens and their identification is based on their growth morphology, characteristic surface antigens, and analysis of their multidirectional differentiation potential. According to the International Society for Cellular Therapy definition, MSCs have the following characteristics: Grow in vitro in culture in an appressed wall with a fibrous cell morphology; express CD44, CD73, CD90, CD105, and do not express the hematopoietic and endothelial cell markers CD45, CD34, CD14, CD11b, CD79α, CD19, HLA-DR; it has the potential to differentiate into osteocytes, adipocytes, chondrocytes, and other multidirectional cells [38, 39]. MSCs are widely distributed in different tissues and can be isolated and cultured in tissues and organs other than bone marrow, such as fat, pancreas, skin, muscle, placenta, umbilical cord, liver, lung, synovial membrane, and UCB. Currently, MSCs that promote hematopoiesis are mainly derived from bone marrow, however, with the increase of age, the number of MSCs and their ability to proliferate and differentiate decreases, limiting their application. Therefore, the search for MSCs from other tissue sources is of great scientific and clinical importance.

First, we take advantage of this to make skeletal muscle a potentially rich source of stromal cells because it is widespread throughout the body and easy to extract. In this study, CD146^+^ PCs (CD146^+^CD56^−^CD34^−^CD144^−^CD45^−^) and MECs (CD56^+^CD34^+^CD144^+^CD45^−^) were successfully isolated and sorted from human skeletal muscle tissue using multiparametric flow cytometry, and the back test purity reached 92.55% ± 0.55% and 96.60% ± 1.14%. The sorted CD146^+^ PCs and MECs were cultured in vitro and grew adherently, the primary cells grew slowly. In short shuttle shape and irregular shape, the cells gradually increased around 7 days; on the other hand, in long shuttle shape, triangular shape, and other irregular shapes, the cell fusion reached 80%–90% after 10–14 days, which were more common in long shuttle shape. The cell morphology was more homogeneous, growing in a swirling, or parallel arrangement. The cells grew rapidly and can generally be passaged within 3–4 days. The above characteristics are consistent with the growth characteristics of MSCs. For the surface markers of CD146^+^ PCs and MECs, we detected them by flow cytometry, and the results showed that both of them highly expressed MSCs markers such as CD44, CD73, CD90 and CD105 and did not express markers CD45 and CD31, which were consistent with the surface marker characteristics of MSCs. To further verify the multidirectional differentiation potential of CD146^+^PCs and MECs, osteogenic, lipogenic, chondrogenic, and myogenic induced differentiation were performed in this study. These results showed that CD146^+^ PCs and MECs sorted by multiparametric flow cytometry had the same cell morphology, immunophenotype, and multidirectional differentiation potential as bone marrow MSCs. Therefore, CD146^+^ PCs and MECs were well isolated, purified, and expanded in vitro in our laboratory, and their easy access, noninvasive to the organism, and high proliferation capacity in vitro can be used as in vitro culture stromal cells for HSPCs.

CD34^+^ bone marrow cells are used in transplantation and gene therapy, advancing research in hematopoiesis. Notably, immunoaffinity-purified CD34^+^ marrow and cord blood cells are rich in CFUs [15].The results of the change in the number of cells before and after coculture in this study showed that the number of CD34^+^ cells in UCB of the culture system with CD146^+^ PCs and MECs as the trophoblast was expanded after coculture for first week and reached the peak at the second week. With the extension of coculture time, the number of CD34^+^ cells in UCB gradually decreased. Compared with the positive control culture system of human BM-MSCs as the trophoblast under the same conditions, the number of cells cocultured at 1, 2, and 4 weeks was not significantly significant (*p* > 0.05, *n* = 5). Compared with the blank control group, the number of cells cocultured at 1, 2, and 4 weeks, was significantly significant (*p* < 0.01, *p* < 0.001, *n* = 5). The results showed that CD146^+^ PCs and MECs increased the survival rate and survival time of HSCs and CD146^+^ PCs and MECs had the similar effect of supporting CD34^+^ HSPCs cells in UCB in vitro as human BM-MSCs. It further confirmed the conclusion put forward by Corselli et al. [10, 11] that PCs have the ability to maintain regeneration and self-renewal potential of HSPCs in vitro.

In terms of colony formation, CD146^+^ PCs, MECs, and human bone marrow MSCs can support the expansion of colony forming cells in UCB under coculture conditions. Moreover, the number of granulocyte macrophage colony forming units of 1, 2, and 4 weeks hematopoietic cells cocultured in the culture system with CD146^+^ PCs and MECs as trophoblast was not significantly different from that in the positive control culture system with human bone marrow MSCs as trophoblast (*p* > 0.05, *n* = 5). These results indicate that CD146^+^ PCs and MECs have the ability to maintain high proliferative potential stem/progenitor cells for a long time similar to bone marrow MSCs.

Hematopoietic cell markers were detected by coculture of CD146^+^ PCs, MECs, and BM-MSCs as trophoblast culture system for 1, 2, and 4 weeks, respectively. The CD45^+^ expression rate of UCB CD34^+^ cells was higher in the CD146^+^ PCs group than in the positive control group at week 2 of coculture and the CD10^+^/CD19^+^ expression rate of UCB CD34^+^ cells was lower in the CD146^+^ PCs and MECs groups than in the positive control group at week 4 of coculture, with statistically significant differences (*p* < 0.05, *n* = 5). The other subtypes of immunophenotypes CD34^+^CD33^−^ and CD14^+^ expression in each group were not significantly different at 1, 2, and 4 weeks (*p* > 0.05, *n* = 5).

Cytokines are the first drugs used for HSCs expansion in vitro and it has been demonstrated that multiple cytokines are able to affect the number of mouse HSCs in vivo, and hematopoietic-related cytokines can promote HSPCs differentiation, proliferation, and directed maturation in vitro and in vivo [40]. MSCs play a pivotal role in hematopoietic engraftment and tissue repair during stem cell transplantation. Furthermore, they regulate the hematopoietic process through the secretion of growth factors and cytokines. The expression levels of cytokines in the culture supernatants of CD146^+^ PCs group, MECs group, and BM-MSCs group were determined by ELISA. The results showed that CD146^+^ PCs and MECs could secrete a variety of hematopoietic related factors, including positive hematopoietic factors, such as early hematopoietic factors SCF and IL-6, to promote the proliferation, differentiation, and mobilization of HSCs [41, 42]. The metaphase hematopoietic factors, such as IL-3 and MCSF, mainly promote the proliferation and differentiation of pluripotent progenitor cells to directional progenitor cells. Thrombopoietin (TPO), also known as megakaryocyte growth and development factor (MGDF), is also expressed in small amounts in striated muscle and bone marrow stromal cells. It can stimulate the secretion and differentiation of megakaryocytes to form a large number of platelets. CD146^+^ PCs and MECs can secrete cytokines required for vascular regeneration, such as VEGF. In addition, CD146^+^ PCs and MECs also secrete some negative hematopoietic factors, such as IFN-γ and TNF-α, mainly block the entry of primitive HSCs into the cell cycle and makes them stay in the stationary phase of the cell cycle. IFN-γ has the antitumor properties of immune modulator, and TNF-α can also inhibit tumor cells by virtue of the immune function of the body [43]. Although there are differences in the expression levels of the above cytokines between CD146^+^ PCs, MECs, and human BM-MSCs groups, there is no statistical difference in the number of cells, colony-forming ability and phenotypic changes of blood cells at each time point of coculture in combination with the three culture systems, indicating that most cytokines cannot act alone and need to work together with other cytokines to regulate the hematopoietic function. In addition, the role of cytokines is concentration-dependent, and some cytokines only have high expression levels; however, it has not achieved specific hematopoietic regulation, so there is no difference in blood cell phenotype. Therefore, our conclusions are consistent with previous studies on the important role of cytokines in differentiation.

In summary, this study found that CD146^+^ PCs and MECs from human skeletal muscle have biological properties similar to those of human bone marrow MSCs, and moreover, CD146^+^ PCs and MECs provide a microenvironment for UCB CD34^+^ cells as a trophoblast, which can regulate the growth, development, and differentiation of hematopoietic cells through direct contact with hematopoietic cells and secretion of hematopoietic-related factors and have the same effect as bone marrow MSCs on human UCB CD34^+^ cells in vitro. Therefore, CD146^+^ PCs and MECs are potentially good stromal cells for in vitro expansion of human UCB CD34^+^ cells and can provide a rich source of stromal cells for the expansion of HSPCs, which will provide a new idea of stromal cell sources for future applications of HSCT.

## 5. Conclusion

In the present study, we investigated the biological characteristics of CD146^+^ PCs and MECs, which isolated and cultured from human skeletal muscle, and further their supporting effect on UCB CD34^+^ cells in vitro. The results showed that CD146^+^ PCs and MECs were consistent with BM-MSCs from cell surface markers and have a good ability of multidirectional differentiation into adipocytes, osteoblasts, and chondrocytes after induction. By establishing in vitro culture system of UCB CD34^+^ cells, UCB CD34^+^ cells were cocultured with human CD146^+^ PCs and MECs as matrix, respectively, and BM-MSCs for feeder layer as positive control at same times. The data demonstrated that there was no significant difference in the number of cells, immunophenotype of blood cells, and colony-forming ability of HSPCs among the coculture system with CD146^+^ PCs, MECs, and BM-MSCs as the trophoblast, so CD146^+^ PCs and MECs as the trophoblast also have certain hematopoietic promotion effect in vitro. They can regulate the growth, development, and differentiation of hematopoietic cells through direct contact with hematopoietic cells. Like BM-MSCs, they have in vitro supportive effects on human cord blood CD34^+^ cells. Therefore, as stromal cells, CD146^+^ PCs and MECs found in this study both were the potential matrix cells for expanding UCB CD34^+^ cells in vitro. In summary, CD146^+^ PCs and MECs from human skeletal muscle have similar biological characteristics to human BM-MSCs, and as the feeder cells, CD146^+^ PCs and MECs provide a microenvironment for cord blood CD34^+^ cells in vitro.

## Figures and Tables

**Table 1:** Culture of CD146^+^PCs and MECs.

Name	CD146^+^PCs	MECs
Culture plate	Inoculated in collagen-coated 96-well plates for adherent culture	

Primary culture medium	DMEM (high glucose) + 20% fetal bovine serum (FBS) + 1% penicillin/streptomycin (P/S)	DMEM (high glucose) + 10% FBS + 10% horse serum (HS) + 0.5% chicken embryo extract (CEE) + 1% P/S

Primary culture method	Adherent culture: The first time 5 days later, half of the medium was replaced. After that, the medium was replaced at an interval of 1 day and the culture was cultured for 14–21 days	

Cell amplification	The cells were digested with TrypLE Express digestive enzyme when the fusion reached 70%–80%, change medium at an interval of 1 day	

**Table 2:** Myoendothelial cells and perivascular stem cells sorted from human skeletal muscle tissues (*n* = 18).

Name	CD146^+^ PCs	MECs
Surface markers	CD146^+^CD45^−^CD34^−^CD144^−^CD56^−^	CD56^+^CD34^+^CD144^+^CD45^−^
Positive rate (%)	0.32 ± 0.09	0.04 ± 0.01
Count (×10^4^)	3.36 ± 0.66	1.26 ± 0.32
Purity (%)	92.55 ± 0.55	96.60 ± 1.14
Survival rate (%)	95.25 ± 0.77	95.68 ± 1.20

## Data Availability

All data generated or analyzed during this study are included in this published article. The data are available from the first author upon request.

## Nomenclature

HSPCs: Hematopoietic stem/progenitor cells
MSCs: Mesenchymal stem cells
MD-PCs: Muscle-derived pericytes/perivascular cells
MECs: Myoendothelial cells
BM-MSCs: Bone morrow-derived mesenchymal stem cells
BMMSCs: Bone marrow stromal cells
SCs: Satellite cells
SP: Side populations
MDSCs: Muscle-derived stem cells
MAPCs: Multipotent adult progenitor cells
SPs: Side populations cells
CB: Cord blood
MP-FACS: Multiparameter flow cytometry sorting technique
CFU: Colony-forming units
HPCs: Hematopoietic progenitor cells
HSCT: Hematopoietic stem cell transplantation
HSCs: Hematopoietic stem cells
hAD-PCs: Human adipose-derived pericyte/perivascular cells.
